# The Changing Epidemiology of Hepatocellular Carcinoma :  Experience of a Single Center

**DOI:** 10.1155/2020/5309307

**Published:** 2020-02-27

**Authors:** Lydia Giannitrapani, Maddalena Zerbo, Simona Amodeo, Elisa Pipitone, Massimo Galia, Tancredi Vincenzo Li Cavoli, Maria Giovanna Minissale, Anna Licata, Cosima Schiavone, Giuseppe Brancatelli, Giuseppe Montalto, Maurizio Soresi

**Affiliations:** ^1^Department of Health Promotion Sciences, Maternal and Infant Care, Internal Medicine and Medical Specialties (PROMISE), University of Palermo, Palermo, Italy; ^2^Department of Biomedicine, Neuroscience and Advanced Diagnostic (Bi. N. D.) Section of Radiological Sciences, University of Palermo, Palermo, Italy; ^3^Unit of Internistic Ultrasound, Department of Medicine and Science of Aging, G. D'Annunzio University, Chieti, Italy

## Abstract

**Aims:**

To analyze the main etiological factors and some clinical features of patients with hepatocellular carcinoma (HCC) at diagnosis and to compare them with those we described ten years ago. *Materials and Methods*. We compared two groups of patients with HCC, Group 1 consisting of 132 patients (82 M, 50 F) diagnosed in the 2003–2008 period and Group 2 including 119 patients (82 M, 37 F) diagnosed in the 2013–2018 period. For all patients, age, sex, viral markers, alcohol consumption, serum alpha-fetoprotein (AFP) levels, and the main liver function parameters were recorded. The diagnosis of HCC was based on AASLD, EASL guidelines. The staging was classified according to the “Barcelona Clinic Liver Cancer staging system” (BCLC).

**Results:**

Mean age was 69.0 ± 8 years in Group 1 and 71.0 ± 9 in Group 2 (*P* < 0.05). HCV subjects were significantly older in Group 2 (*P* < 0.05). HCV subjects were significantly older in Group 2 (*P* < 0.05). HCV subjects were significantly older in Group 2 (*P* < 0.05). HCV subjects were significantly older in Group 2 (*P* < 0.05). HCV subjects were significantly older in Group 2 (*P* < 0.05). HCV subjects were significantly older in Group 2 (*P* < 0.05). HCV subjects were significantly older in Group 2 (*P* < 0.05). HCV subjects were significantly older in Group 2 (

**Conclusions:**

This study shows that over the last decade a number of features of patients with HCC in our region have changed, particularly age at onset, etiological factors, and staging of HCC.

## 1. Introduction

Hepatocellular carcinoma (HCC) is a major health problem, as in 2016 one million incident cases of liver cancer globally and 829,000 deaths were recorded. It ranks as the fifth most common cause of cancer in men and the seventh in women [1] representing a third of all cancer-related deaths and the leading cause of death in patients with liver cirrhosis [1–3]. The causes of this tumor coincide with those of liver cirrhosis, although there are epidemiological differences according to the geographical area considered. In detail, hepatitis B virus (HBV) etiology is prevalent in China, Southeast Asia, and sub-Saharan Africa [4, 5], while chronic hepatitis C virus (HCV) is an important risk factor in western countries and Japan [6–8]. Chronic alcoholic liver diseases are reported worldwide, with the highest prevalence in Eastern and Central Europe (53 and 46%, respectively), sub-Saharan Africa (40%), and North America (37%) [9]. The role of aflatoxins is greater in Africa and Eastern Asia [4]. Although nonalcoholic steatohepatitis (NASH), autoimmune, and cholestatic diseases predispose to HCC onset, they seem to play a minor role [10]. However, in recent years, the epidemiological scenario has been changing: there is a lower severity of the disease at diagnosis [7], a reduction in HCC incidence in areas with a traditionally high prevalence, such as East Asia, but an increase in low prevalence areas [11–13]. Indeed, in geographical areas with the highest HCC incidence, such as China and Eastern Sub-Saharan Africa, a reduction in the number of new cases of the disease was observed in the 1990–2015 time interval, probably attributable not only to vaccination programs but also to a lower exposure to the predominant causal factors in these areas, such as aflatoxins [14]. Studies in the United States, Canada, Australia, New Zealand, and much of Europe, and also the Philippines and Guatemala, have shown increases in cases of HCV and alcohol-related HCC of 42% and 56%, respectively [1, 3, 14–22]. In western countries, there is an increasing incidence of post-NASH HCC [11, 12, 22–24]. The use of direct antivirals (DAAs) may also affect HCC epidemiology in the coming years. HCC prognosis, however, remains poor [9] and especially in Italy, where the 5-year survival rate is 20%, with a north-south gradient (i.e., it is worse in the southern regions), despite the continuous surveillance programs [11, 12, 25]. Moreover, Sicily is a region where the relationship between chronic viral infections and tumors is particularly high; it is an example of the association between *Helicobacter pylori* (*H. pylori*) and *Epstein-Barr virus* (*EBV*) and gastric cancer [26], or between EBV and rhinopharyngeal tumors [27]. As regards epidemiological features of HCC, Sicily has also some peculiarities between those of western and southern countries: HCV prevalence is greater than that in the rest of Italy, alcohol consumption is less important, there are high migratory flows of populations from geographic areas with a high HBV endemicity, and the survival rate is lower than that in other Italian regions [7]. The aim of our study was to analyze the etiology, clinical presentation, and staging of HCC diagnosed at our center between 1^st^ January 2013 and 31^st^ December 2018 and to compare the results with those observed between 2003 and 2008 at our Department of Clinical Medicine in Palermo and already analyzed in a previous study of us [7].

### 1.1. Patients and Methods

Group 1 included 132 patients (82 M/50 F) diagnosed with HCC in the period between 2003 and 2008. In Group 2, there were 119 patients (82 M/37 F) with HCC diagnosed between 2013 and 2018. For all patients, the following data were recorded at HCC diagnosis: age, sex, and the data from a dedicated standard questionnaire investigating the presence of diabetes mellitus, arterial hypertension, cardiovascular disease, the use of any hepatotoxic drugs, and alcohol consumption. Serum markers of hepatitis B and C viruses, anti-HDV (in HBsAg positive subjects), serum AFP levels, and the main parameters of liver function, serum ferritin, and iron levels were assayed by standard commercially available kits with an automated analyzer. In the absence of a viral or alcoholic pathology, autoantibodies were also assayed: antinuclear (ANA), antismooth muscle (ASMA), anti-Microsomal Liver Kidney Microsome-1 (anti-LKM1), antimitochondrial (AMA), perinuclear antineutrophil cytoplasmic (pANCA), and antitrypsin alpha-1.

### 1.2. HCC Diagnosis

Group 1 patients were diagnosed in accordance with the AASLD 2005 criteria [28], while in Group 2 patients the EASL 2012 and AISF 2013 guidelines criteria were followed [29, 30]. HCC staging was assessed using the Barcelona Clinic Liver Cancer staging system (BCLC) [31, 32]. Histology: the diagnosis was histological in 55% of the Group 1 patients and in 9.2% of the Group 2 patients.

### 1.3. Liver Cirrhosis Diagnosis

In both periods, liver cirrhosis diagnosis was based on histological findings or unequivocal clinical and biochemical signs, associated with at least one positive imaging technique (ultrasound or computerized tomography). Only in 27% of the patients in Group 2 was elastography (Fibroscan Echosens) also used. The staging of cirrhosis was based on the Child-Pugh score [33].

### 1.4. Alpha-Fetoprotein

Alpha-fetoprotein (AFP) values >200 ng/ml were considered diagnostic, in accordance with the AASLD 2005 guidelines. AFP values were also classified according to the following cut-offs: 0–20 ng/ml, 21–200 ng/ml, 200–400 ng/ml, and >400 ng/ml. The 20 ng/ml cut-off was used since this is the limit value considered by the method kit, 200 ng/ml is the cut-off considered diagnostic by the AASLD 2005 guidelines, and 400 ng/ml is the cut-off in the EASL 2001 guidelines.

### 1.5. Classification Based on Viral/Nonviral Etiology

1. HCV: anti-HCV positive patients
2. HBV: if patients were positive for HBV surface antigen (HBsAg) (only one patient was anti-HDV positive)
3. HBV/HCV: if they were positive for both HBsAg and anti-HCV
4. Nonviral: when HBsAg and anti-HCV virus markers were absent

Nonviral patients were divided into

- Alcoholic: if the daily intake of ethanol was >40 g for women and >30 g for men, for more than 10 years, in the absence of other causes of liver damage
- Post-NASH: in accordance with the AASLD [34] and defined metabolic guidelines
- Other etiologies: including hemochromatosis, Wilson's disease, antitrypsin alpha-1 deficiency, primary biliary cholangitis, and sclerosing cholangitis
- Cryptogenic: if patients were not positive for HBsAg or anti-HCV antibodies, alcohol abuse, autoimmune, or genetic liver diseases. Finally, if the cryptogenic patients had an associated history of arterial hypertension, diabetes or obesity were included in this metabolic group.

Seven Group 2 patients had a mixed HCV and alcoholic etiology.

### 1.6. Statistical Analysis

Continuous variables are expressed as mean ± standard deviation, and dichotomous variables (present/absent or yes/no) as number and percentage. To evaluate differences in the means and frequencies between the two groups, Student's *t*-test, *χ*^2^ test, and Fisher's exact test were used. Spearman's rank correlation coefficient was used where appropriate. The data were significant if *P* < 0.05.

## 2. Results

Table 1 shows the demographic features of the two groups, analyzed both globally and also divided according to etiology.

Male sex was prevalent in both groups, but without a statistically significant difference (61.1% versus 69%, *P*=ns). The average age, for all causes, was significantly higher in the Group 2 patients (*P* < 0.05). When age was assessed in relation to etiology, HCV patients in the 2003–2008 period were younger (*P* < 0.05). In HBV patients, on the other hand, the mean age in Group 2 was significantly lower (68.4 ± 3.1 versus 57.7 ± 17 years; *P* < 0.04). In the second group, four patients were immigrants from non-European countries (3 from sub-Saharan Africa, 1 from Pakistan). After eliminating these 4 patients from the group data, the average age of HBV in Group 2 rose to 67 ± 7.2 years and was similar to the one of Group 1 (*P*=ns). The age of the immigrant HBV patients ranged between 16 and 40 years, and none of them were consuming alcohol. In Group 1, there were no immigrants.

**Figure 1:**
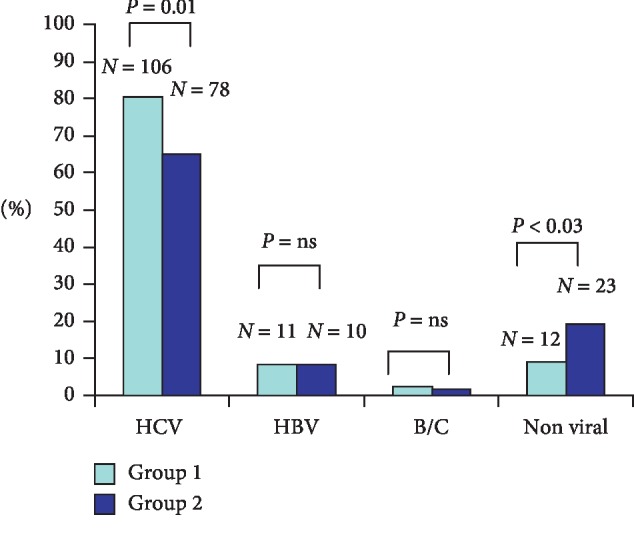
Comparison of the prevalence of etiologies in the two periods.

When we compared the prevalence of HCV-related HCC in the two periods, it was found to be significantly lower in Group 2 than in Group 1 (65% versus 80.3%, respectively, *P*=0.01). On the other hand, nonviral HCC was significantly higher in Group 2 versus Group 1 (17% versus 9%; *P* < 0.03) (Figure 1).

In both periods, HBV prevalence was 8.3%, although after removing the immigrant patient data from the second period, prevalence dropped to 5.8%, but without reaching statistical significance.

**Figure 2:**
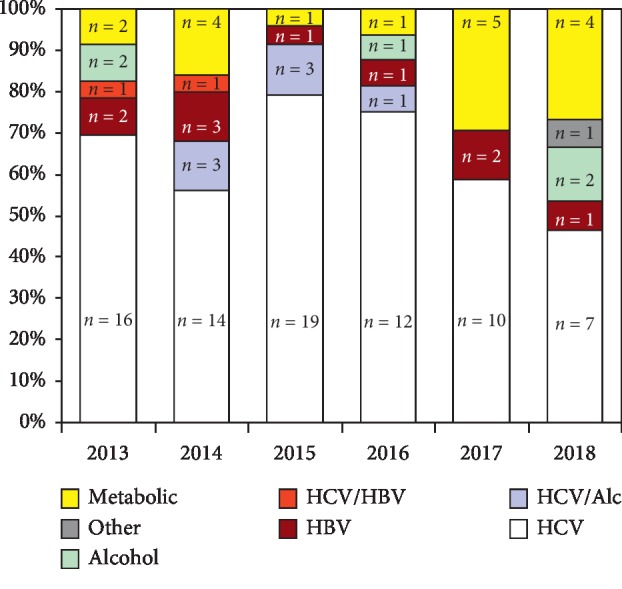
Incidence of HCC divided according to etiology.

Figure 2 shows the annual incidence of HCC divided according to etiology. We observed a progressive reduction in HCV etiology, which fell from 70% in 2013 (with a peak of 79% in 2015) to 47% in 2018, as well as an increase in cryptogenic/metabolic forms. The limited number of cases, however, did not allow us to evaluate any differences in HCC incidence in the other etiologies. By contrast, HBV etiology, which was the most numerous among these, appeared constant in both periods.

**Figure 3:**
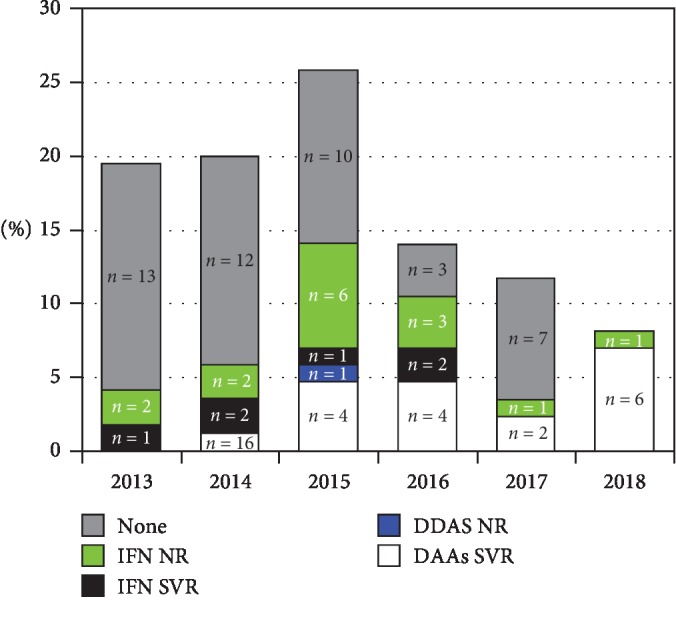
Incidence of HCV-related HCC from 2013 through 2018, antiviral therapy used, and type of response (NRs or SVRs).

**Figure 4:**
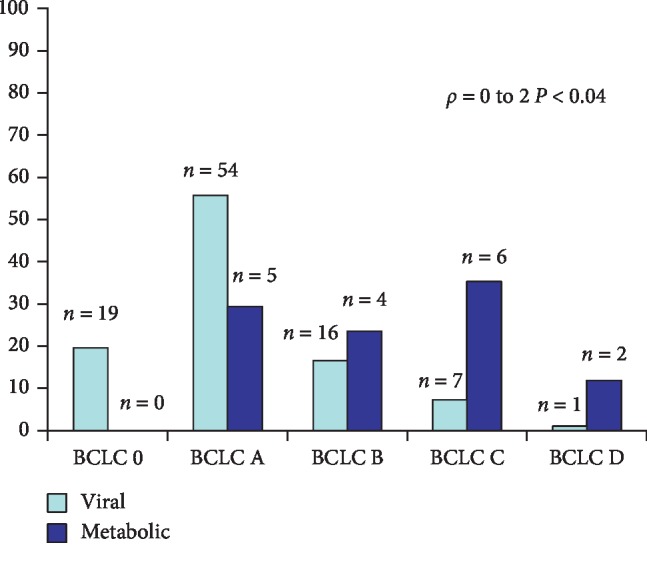
BCLC staging at HCC diagnosis for viral versus metabolic/cryptogenic etiology in the years 2013–2018.

Figure 3 shows the annual incidence of HCV-related HCC from 2013 through 2018, the antiviral therapy used, and the type of response, with patients being divided into nonresponders (NRs) or sustained virological responders (SVRs). The antiviral therapies were interferon, if commenced before 2013, or directly acting antivirals (DAAs). Figure 3 also includes patients with mixed etiology (HCV + alcohol). It can be seen that the annual incidence of HCV-related HCC etiology progressively decreased from 2015 through 2018 and that HCC also occurred in patients who had previously been SVRs to both INF and DAA therapy. Only one of the HCC patients treated with DAAs was an NR.  Table 2 compares the staging of cirrhosis underlying hepatocellular carcinoma according to the Child-Pugh classification in the two study groups. In the Group 2 patients analyzed globally, the Child-Pugh score showed no statistically significant differences (*P*=ns). When evaluating HCV patients, there was a trend towards a less severe Child-Pugh in Group 2 but without reaching any statistical significance. In contrast, in patients with nonviral etiology, in Group 2, there was greater severity but once again not significant.  Table 3 shows the staging according to the BCLC score in the two groups analyzed globally and divided by etiology. At the time of diagnosis in Group 2, the staging of HCC is less severe than in Group 1 both in the total population (*ρ* = −0.16, 0.002) and in subjects with HCV etiology (*ρ* = −0.29, 0.0001). The group of nonviral HCCs showed no statistical differences between the two periods (*ρ* = −0.13; *P* = ns). In patients with HBV etiology, in Group 2, the staging was more severe (*ρ* = 0.25; *P* = ns). In the second period compared to the first, the diagnosis in patients subjected to surveillance was significantly greater: 98/120 (60%) versus 93/132 (70%), *P* < 0.05.  Figure 4 compares the BCLC staging at HCC diagnosis for viral versus metabolic/cryptogenic etiology in the years 2013–2018. In nonviral forms, BCLC staging was more severe (*r* = 1.9, *P* < 0.01). Assuming an alpha-fetoprotein value >200 ng/ml as diagnostic, HCCs with AFP greater than this cut-off in Group 1 were 30/132 (22.7%) and in Group 2 20/208 (9.6%), *P* < 0.002. Finally, Table 4 compares the frequency of AFP at various cut-offs between metabolic and viral HCCs. It is noteworthy that in viral forms AFP values are more frequently higher than 200 ng/ml (*ρ* = 0.3, *P* < 0.0001).

## 3. Discussion

Our study reports the experience of a single center, which in the past had already reported some epidemiological data about HCC in a specific geographical area, Sicily, where some aspects are known to be different from Italy as a whole, and in an intermediate situation between western and southern world countries.

### 3.1. Age and Gender

The increase in the average age of HCC at diagnosis is a finding already reported in the literature [8, 11, 25], and our group had also detected a significant increase in age in a previous study conducted on a Sicilian population, comparing the epidemiology of the nineteen-nineties with those of the first decade of the twenty-first century [7].

In this case study, HCC diagnosis was also made in older patients: in Group 2, age at diagnosis was significantly higher than in Group 1, both in the entire study population and in the HCV etiology cases alone. Age was higher even in the nonviral etiology patients of the second group, but the difference was not statistically significant.

The reasons for the progressive aging of the HCV-related HCC population are to be correlated to the older age of patients with cirrhosis and depend on the limited number of new infections occurring in the last few decades [35]. Indeed, following the discovery of the HCV virus in 1989, and thanks to prevention strategies, HCV circulation has been reduced, thus limiting new infections in younger generations [35], and the future effects of eradicating HCV infection by direct-acting antiviral (DAA) therapies will further improve this situation [36].

An interesting result concerned the HBV-related HCC patients. Their average age in the second group was significantly lower, but the difference was no longer significant when we calculated it after removing the immigrant data. The age of the non-EU patients with HCC ranged from 18 to 40 years and they came from sub-Saharan Africa and Asia, areas where HBV incidence is high [14].

During both observation periods, the frequency was higher in males, with an overlap in prevalence between the two groups, confirming that it is the sex most frequently suffering from HCC.

### 3.2. HCC and Migration Flows

The results of the epidemiology of HBV-related HCC patients in our study highlight the impact that migration flows have had and will continue to have over the coming years on the presentation of HCC, its prognosis, and more generally the etiology of liver disease in Italy.

In recent years, migration from African countries, and to a lesser extent from Asia, has been increasing, especially towards Sicily. In the past, we helped to outline the Sicilian epidemiological profile of the diseases these migrant populations suffer from, showing that after infectious diseases, neoplasia is the second most frequent cause of hospitalization and recourse to “day service” or “day hospital” treatment. We also reported that HBV is the most frequent etiology in the context of liver disease, in agreement with the literature data [37, 38]. In our series, the average age of HCC in the immigrants was about 30 years, suggesting a vertical transmission of the infection, probably at birth. These are patients from highly endemic HBV geographical areas, where vaccination programs have only recently been implemented or are still lacking [14]. Furthermore, two of these patients were in BCLC stage B and one in stage C, indicating that the diagnosis was not early (data not shown). This underlines the need to launch appropriate screening programs in the immigration centers and health facilities that care for these patients, in order to permit early diagnosis of HCC and to identify patients with chronic HBV-related liver disease which, with appropriate treatment, can help eliminate the HBV reservoir in these migrant populations.

### 3.3. Etiology

As in other models of carcinogenesis linked to chronic infections [39], the correlation with HCV and HBV-related chronic liver diseases remains important, even if we have detected relevant epidemiological variations. In both periods, the most frequent cause of HCC was HCV, with a significant reduction in prevalence in the second period compared to the first (65% versus 80%). This decreasing trend in HCV etiology in HCC patients in Italy had already been reported in 2010 by Stroffolini in an Italian multicenter study [40], but, in the same year, a similar study carried out in Sicily reported an increase in the prevalence of HCC-related HCV etiology [7]. This difference in trend was probably due to the higher prevalence of HCV-related liver disease in Sicily, which would explain the later inversion of the trend highlighted by the results of this study, which are in agreement with the national epidemiological trend pointing to a recent progressive reduction in the etiologic role of HCV from 71.1% in 1996 to 57.2% in 2014 [8, 11, 41]. The significant drop in HCV etiology over the past 5 years may also depend on the introduction of DAA therapy, which can eradicate HCV in more than 90% of cases [36]. The data in the literature on the role of DAAs in HCC onset is conflicting. It is reasonable to expect HCV eradication to result in a reduction in HCC incidence in sustained viral responders (SVRs), similarly to what studies on the use of interferon have shown [42, 43]. However, despite these assumptions, some studies have surprisingly reported an increased risk of HCC after therapy with DAAs [44–46]. It should be emphasized that most of these studies were retrospective, not multicenter, they did not compare SVRs on DAA treatment with untreated HCV patients, and only a few of them compared the occurrence rate of post-DAA HCC patients with groups on interferon therapy. Other studies, on the other hand, have reported that there is a significantly lower incidence of HCC in SVRs to DAA treatment than in NRs and an incidence similar to SVRs receiving interferon antiviral therapy [47–49]. Although our study was not designed to demonstrate the effect of DAAs on HCC and despite the small number of cases, our data would appear to confirm the positive effect of this therapy, as supported by the progressive reduction in the prevalence of HCV etiology in HCC after 2015, just 2 years after DAA began to be used (in 2014). The onset of HCC in post-DAA SVR cirrhotic patients (only one of our patients was an NR) also confirms that screening for HCC should be lifelong in these subjects. In contrast to the decline in HCV etiology, our results show that nonviral etiologies, especially metabolic ones, have increased. Bucci et al. recently reported that between 1996 and 2014 the prevalence of HCC with NAFLD/cryptogenic etiology increased from 1.2% to 12.6% and that the prevalence of nonviral causes—multiple etiologies—rose from 0.02% to 4.9% [11]. In our study, the metabolic forms increased for two reasons: on the one hand, due to a real increase, as underlined by the number of cases (3 times higher than in the first period), and on the other, due to a relative increase following the reduction in HCV as the main cause of HCC. A final comment should be made on HBV etiology. A comparison between the two periods shows a virtually identical prevalence of 8.3%. However, if the immigrant data are removed, prevalence falls to 5.3%. This data is in line with reports from Italy as a whole and other western countries and depends both on the progressive reduction in HBV etiology as a result of vaccination campaigns and on the use of antiviral drugs which, despite not being able to completely eradicate the virus, reduce its activity and thus the incidence of new HCCs [41].

### 3.4. Liver Cirrhosis Severity and HCC Staging

In the second period, unlike the first, we found 2 cases of HCC without cirrhosis, which is in line with studies in the literature that report this possibility in about 10% of cases.

### 3.5. Child-Pugh Score

In our series, we did not find significant differences in the Child-Pugh score between the two periods. This likely depends on the contrasting behavior patterns of viral and nonviral cirrhosis and on the limited number of cases. In the HCV-related HCC patients, Group 2 had a less severe, although not statistically significant CP score; we did observe an increase in HCC diagnoses with Child-Pugh A from 67.4% to 75.3%, which is comparable to the gradual increase in Class A between 1996 and 2014 recently reported by Stroffolini et al. [41].

### 3.6. BCLC Score

Comparing the BCLC Staging System scores in the two groups, in the second period, we found that staging was significantly less severe. It is likely that the better staging in Group 2 depends on the larger number of patients on six-monthly ultrasound surveillance.

### 3.7. Comparison of HCC Staging between Viral and Metabolic/Cryptogenic Etiology

The patients with metabolic/cryptogenic cirrhosis had a more severe BCLC staging than the viral etiology patients, and only 8/25 (32%) were aware they were suffering from chronic liver disease and were undergoing periodic ultrasound scans; therefore, finding a neoplasm with a more severe staging in these subjects may be due to a lack of careful surveillance, as already reported in the literature [11, 23, 50]. These results, in agreement with the increase in the prevalence of the cryptogenic/metabolic forms, open a discussion on the need to extend ultrasound screening for HCC. All the AASLD, EASL, APASL, and AISF guidelines recommend that cirrhosis patients (CP Classes A and B) should be screened for HCC semiannually [9, 28, 51, 52]. Surveillance is performed with ultrasound, but it has limitations: on one hand, due to its reduced sensitivity in cases of obesity, and on the other hand, due to the possibility that HCC may occur in noncirrhotic liver, especially in patients with NASH [53]. For this reason, caution is required in planning screening and in extending it to all patients with steatosis on ultrasound. The lack of reliable data in these cirrhotics and even more so in noncirrhotic patients makes it difficult to develop screening policies that can optimize the cost/benefit ratio. Recently, the Italian Association for the Study of the Liver proposed a flow-chart using clinical, elastography, and ultrasound data to select patients with hepatic steatosis at risk of evolution as candidates for regular surveillance [54].

### 3.8. Alpha-Fetoprotein

The use of AFP in HCC diagnosis is questioned by western guidelines, as opposed to those in Asia where it continues to play a role [9, 52, 53]. In our study, the reliability of AFP in defining HCC was limited, especially in the second period. An explanation of this difference between the two periods could be the greater dimensions of the neoplasm in the Group 1 patients. Previous studies, in fact, have found that AFP levels correlate positively with TNM staging, which can be explained by the fact that neoplasm size can affect AFP levels in two ways: larger masses can (1) secrete higher amounts of AFP and (2) be made up of different clones and have a higher probability of being AFP producers [55–57]. Our results confirm our past reports; i.e., AFP values are lower in patients with nonviral HCC [54].

## 4. Conclusions

Our study was limited by the small number of cases, which do not allow us to evaluate alcohol, HBV/HCV coinfections, or associated etiological factors for HCC; however, we can observe the following:

1. There has been a reduction in HCV etiology which, however, still remains the most frequent cause of HCC
2. Diagnosis of HCC occurs at an older age, at least in HCV patients
3. DAA therapy will change the prevalence and incidence of HCC in the coming years
4. Prevalence, age, and severity of HCC in HBV patients may be affected in the coming years by migration flows
5. The BCLC-based staging of the tumor at diagnosis and that of Child-Pugh for staging cirrhosis are less severe
6. In HCCs with a metabolic etiology (post-NASH) at diagnosis, BCLC staging is more severe than viral etiology
7. The AFP assay is not very useful, especially in nonviral forms
8. It is necessary to establish accurate surveillance programs in the Immigration Centers in order to make an early diagnosis of HCC
9. Further studies are needed to define screening policies for HCC on NAFLD, which is the second leading cause of disease in our geographic area today, and which will probably become the first in the coming years

## Figures and Tables

**Table 1:** Mean age and M/F ratio of HCC patients of the two periods globally and divided according to etiology.

	Group 1			Group 2			*P*<
	*n*	M/F	Age	*n*	M/F	Age	
	132	82/50	69.0 ± 8.0	120	83/37	71.0 ± 9.0	0.05
HCV	106	62/44	70.1 ± 7.3	78	48/30	72.1 ± 7.7	0.05
HBV	11	10/1	68.4 ± 3.1	10	9/1	67.0 ± 7.2	ns
B/C	3	2/1	71.3 ± 3.3	2	1/1	58.1 ± 10.2	ns
N. vir.	12	8/4	69.0 ± 9.0	7	7/0	70.0 ± 9.01	ns

**Table 2:** Child-Pugh class at HCC diagnosis according to etiology.

	Group 1			Group 2		
	A	B	C	A	B	C
*n*	89	36	7	82	32	6
%	67.4	27.2	5.3	68.3	26.6	5
HCV *n* =	72	29	5	58	18	2
%	68.7	27.3	5	74.3	23.1	3.8
HCV/HBV *n* =	3	0	0	1	1	0
%	100			50	50	
HBV + *n* =	8	2	1	9	1	0
%	72.7	18.2	9.1	90	10	0
Mixed *n* =				4	3	0
%				57.1	46.9	
N. vir. *n* =	6	5	1	10	9	4
%	50	41.6	8.4	43.5	39.1	17.4

**Table 3:** BCLC score staging in patients divided according to etiology.

	Group 1						Group 2					
	BCLC 0	BCLC A	BCLC B	BCLC C	BCLC D	Tot	BCLC 0	BCLC A	BCLC B	BCLC C	BCLC D	Tot
*n* =	10	59	29	24	10	132	19	61	22	15	3	120
%	7.6	44.7	21.29	18.2	7.6		15.8	50.8	18.3	12.5	2.5	
HCV *n* =	10	45	21	21	9	106	16	44	12	5	1	78
%	9.4	42.4	19.8	19.8	8.4		20.5	55.1	15.4	6.4	2.6	
HBV *n* =	0	7	3	1	0	11	1	5	3	1	0	10
%	0	63.6	27.3	12.5	0		10	50	10	30	0	
B/C *n* =	0	2	0	1	0	3	0	1	0	1	0	2
%	0	66.6	0	33.3	0		0	50	0	50	0	
—	—	—	—	—	—		2	4	1	0	0	7
							28.5	57.1	14.3	0	0	
N vir *n* =	0	5	5	1	1	12	0	7	6	8	2	23
%	0	41.6	41.6	8.8	8.8		0	30.4	26.1	34.7	8.7	

**Table 4:** Frequency of AFP at various cut-offs between metabolic and viral HCCs.

	Viral *N* = (%)	Metabolic *N* = (%)
AFP 0–20 ng/ml	63 (64.9%)	15 (83%)
AFP 21–200 ng/ml	14 (15.4%)	2 (17%)
AFP 201–400 ng/ml	10 (8.3%)	0 (0%)
AFP > 400 ng/ml	10 (8.3%)	0 (0%)

*ρ* = −0.3; *P* < 0.0001.

## Data Availability

The datasets used and/or analyzed during the current study are available from the corresponding author on reasonable request.
